# Climate change can affect crop pollination in unexpected ways

**DOI:** 10.1093/jxb/erx075

**Published:** 2017-05-11

**Authors:** Fred L Stoddard

**Affiliations:** Department of Food and Environmental Sciences, University of Helsinki, Finland

**Keywords:** Bee pollination, breeding systems, climate change, crop pollination, faba bean (*Vicia faba*), self-pollination

**Climate change may affect us in more ways than we have anticipated. In this issue of *Journal of Experimental Botany* (pages 2055–2063), Bishop *et al.* show how the mixed breeding system of faba bean, an important protein crop for regions that are too cool for soybean, changes with heat stress from self- to cross-pollination.**

Faba bean (*Vicia faba*) is a valuable crop for many reasons. One of the earliest crops to be domesticated, it has been used in food for at least 10 000 years. Of the starchy legumes, it is highest in protein, with a global average protein concentration of 29% (dry matter basis) – more than common bean, lentil, chickpea, cowpea or common pea (Feedipedia, http://www.feedipedia.org). In many places, either its yield or its protein yield is greater than that of the other starchy legumes (Eurostat, http://ec.europa.eu/eurostat/web/main/home; FAOstat, http://www.fao.org/faostat/en/#home). It grows in cool, moist conditions, such as winters in Mediterranean and maritime climates or summers in cool-temperate climates.

Nevertheless, it has its faults. It has a very large genome, 13 Gbp, more than twice that of pea, so genomic knowledge has lagged behind that of simpler species. It protects itself against herbivores using the same methods as other plants, with the seeds containing trypsin inhibitors, amylase inhibitors, tannins and lectins, but also with a pair of unusual pyrimidine glycosides, vicine and convicine. These cause a haemolytic anaemia, called favism, in susceptible humans and a similar problem in many breeds of chicken. One of the biggest barriers towards progress, however, is its mixed breeding system, which prevents handling it either with pedigree methods as a reliable inbreeder, or with F1 hybrid methods as a self-incompatible or male-sterile out-crosser. Hence the breeding system has been a subject of investigation and experimentation for several decades.

## Beans need bees

The papilionoid legume flower is well adapted for bee-mediated pollination, and the legumes and bees are often said to have co-evolved (Box 1). The plant produces a great excess of flowers, in contrast to pea, lentil or chickpea, so it is adapted to export of pollen.

Box 1. **Bee-mediated pollination**

The faba bean flower has a nectary at the base of the pistil, a sweet scent, and a generous quantity of pollen held at the front. The venation of the keel petal and the black spots on the wing petals of wild-type flowers, also visible in UV, guide the bees. Above, wild *Bombus pascuorum*; below, honeybee. Reproduced, with permission, from Jake Bishop and Jeff Paull, respectively.

**Figure:**
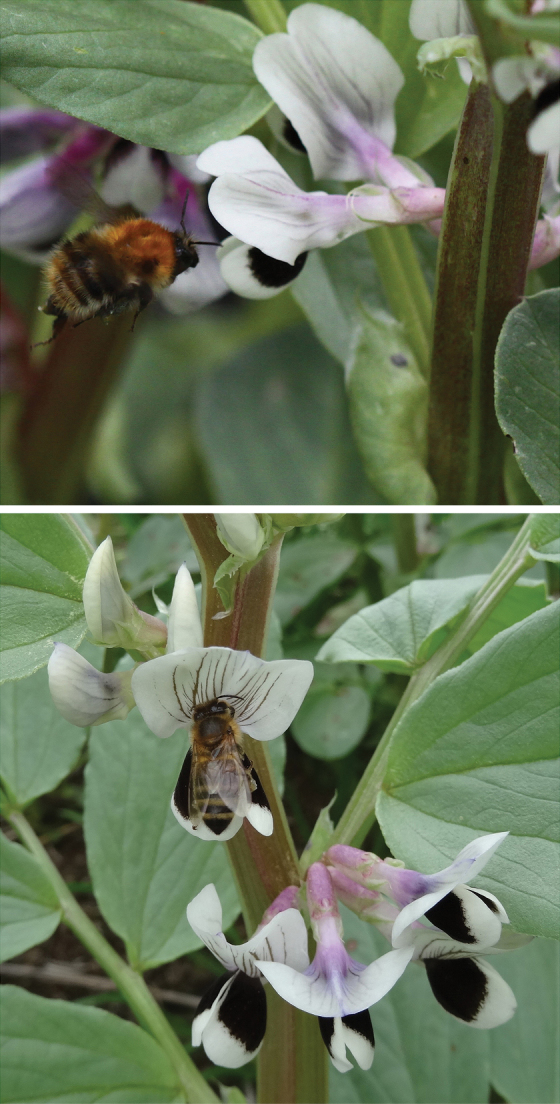

Faba bean workers have termed the ability of the flower to pollinate itself in the absence of bee activity as ‘autofertility’ (Drayner, 1959). Several components of flower structure are considered to affect autofertility, including the volume of pollen, the angle of the style to the ovary, the length of papillae on the stigma, the thickness or hardness of the cuticle that retains the stigmatic exudate, and the presence or volume of that exudate (Chen *et al.*, 2006). The importance of some parts of this package, such as style angle and papilla length, is unclear, but it is clear that the pollen has to reach the stigma, and that the exudate has to be released for pollen to germinate. The activity of a bee in forcing down the wing petal–keel petal complex forces the plug of sticky pollen onto the stigma, then the bee’s action in either pushing into the flower to reach the nectar or gathering (‘scrabbling’) the pollen ensures that the stigmatic cuticle is broken and the exudate is released. Pollen brought on the bee’s body may be from the same flower, the same plant, or another plant (Stoddard and Bond, 1987). Most F1 hybrids are 100% autofertile, as their pollen quantity is great, the stigmatic papillae are short and their stigmatic exudate is copious. Autofertility increases through the life of the plant (Porceddu *et al.*, 1980), possibly because of changes in temperature, daylength or water availability, or from internal regulation.

How, then, does the environment, and potentially climate change, affect this process? Thirty years ago, I looked at the effects of water deficit on autofertility (Stoddard, 1986*a*) in the hot summer conditions of South Australia, with daily temperatures regularly around 40°C. Some plots were given generous trickle irrigation and others limited. Pollen deposition was poorer in the droughted flowers, germination was poorer, and the pollen tubes were less likely to reach the basal ovules. While poor pollen tube growth was at least partly attributable to poor turgor in the droughted plants, other aspects were less easily explained away. The results were partly compatible with the model that water deficit would inhibit the spontaneous rupture of the stigmatic cuticle (Lord and Heslop-Harrison, 1984)

Male function of flowers is notoriously susceptible to environmental stresses, as nicely reviewed by Parish *et al.* (2012). In my experience with various species in conditions of moderate water deficit stress, anthers often fail to dehisce, or pollen quantity is reduced. These problems are easily noticed when one is manually cross-pollinating plants to develop experimental populations, although one seldom bothers to quantify the effect. As Onyemaobi *et al.* (2017) have shown for common wheat, the fault is not only with the male, and female fertility may also be reduced.

Heat stress causes many of the same sorts of oxidative stresses as water deficit stress. Bishop *et al.* (2016) have already quantified the lethal effect of temperatures above 28°C on pollen germination of winter bean. Bishop’s heat-stressed beans may have suffered from other alterations, as listed above, but the most likely are a harder cuticle impeding release of stigmatic exudate and retention of pollen, reduced volume of stigmatic exudate, or inhibited anther dehiscence.

## What about the bees?

What are the implications for the future? Greater reliance on wild pollinators is a risky strategy when their populations will also be affected by climate change. Whether autofertile or not, faba bean is one of the mass-flowering crop species that is known to support the population density of wild bumblebees (Westphal *et al.*, 2003) and solitary bees (Holzschuh *et al.*, 2013). Models of the effects of climate change on European bumblebees show that distributions will move northward in latitude and upward in altitude (Rasmont *et al.*, 2015), and that some species have already done so (Martinet *et al.*, 2015), while others are at greater risk of extinction (Rasmont *et al.*, 2015).

Honeybees cannot reach the nectar of undamaged faba bean flowers, but can gather pollen. In projects as far apart as the UK and Australia, we have noticed that honeybees vary in their effectiveness at working faba bean crops, with some colonies vigorously gathering pollen and others avoiding doing so. Perhaps this is attributable to differences in the colonies’ needs for essential amino acids (Cook *et al.*, 2003) or their sensitivity to the odour of the pollen (Cook *et al.*, 2005).

Thus increased production of faba bean and other grain legumes, as promoted in the European Union’s Common Agricultural Policy revision of 2013 (European Union, 2013), should assist in the maintenance of populations of wild bees (Pywell *et al.*, 2015). In Australia, where there are no native bumblebees, honeybees have become feral. When faba bean was a rare crop in the 1980s, I found that its pollination by feral honeybees was more than adequate (Stoddard, 1991). A more recent study, when its area had increased 2.5-fold, found that yield increased when hives of honeybees were provided (Cunningham and Le Feuvre, 2013). In other latitudes, crops as diverse as British winter beans and Finnish spring beans have shown a clear need for supplemental pollination, as provided by hives of honeybees (Stoddard, 1986*b*; Varis, 1995). Provision of honeybees may be increasingly necessary to ensure adequate pollination of faba bean crops, because of questions about the ability of wild pollinator populations to keep pace with the increasing areas of the crops, combined with the effects of climate change on both those wild pollinators and the pollination system of the crop.

Plant breeders hardly need additional breeding objectives in their already complex programmes, but resistance to oxidative stresses is generally on their agendas. Genomic analysis may help us to identify both the mechanisms by which the heat stress affects self-pollinating ability, and pathways for generalized resistance to oxidative stresses or specific resistance to heat and water deficit. The combination of breeding for resistance to heat and water deficit with management to maximize pollination will ensure the continued productivity of this important crop.
